# Non-falciparum malaria infections in Uganda, does it matter? A review of the published literature

**DOI:** 10.1186/s12936-024-05023-9

**Published:** 2024-07-12

**Authors:** Mansour Ranjbar, Yonas Tegegn Woldemariam

**Affiliations:** WHO Uganda Country Office, Kampala, Uganda

**Keywords:** Severe malaria, Non-falciparum, Mixed infections, *P. ovale* spp*.*, *P. malariae*, *P. vivax*, *P. falciprum*, Uganda

## Abstract

**Background:**

*Plasmodium falciparum* is the dominant malaria species in the sub-Saharan Africa and the main cause of severe disease and death. Notwithstanding, severe malaria and death due to non-falciparum infections have been reported, but at much lower rates than *P. falciparum* infections. Following increasing use of molecular detection techniques in epidemiological studies, a higher prevalence of non-falciparum species has been reported in the region than previously thought. This article reviews the literature on the prevalence of non-falciparum malaria species in Uganda and the clinical figures of their severe diseases. It aims to elucidate the extent to which mono non-falciparum malaria infections in a highly malaria-endemic country contribute to malaria mortality and outline its policy implications on malaria case management.

**Methods:**

The available English-language published peer-reviewed literature up to March 2024 was sought via PubMed and Google Scholar. The keywords used were severe malaria, AND *P. falciparum*, *P. malariae*, *P. vivax*, *P. ovale* spp*.*, mixed infections AND Uganda. The review encompassed 53 articles. Articles using molecular diagnosis methods were accounted for analysis.

**Results:**

The literature reported a substantial prevalence of non-falciparum infections in Uganda. *Plasmodium malariae* and *Plasmodium ovale* spp*.* were the second and third most prevalent reported malaria species respectively after *P. falciparum* as dominant species. Non-falciparum malaria infections often occur as mixed infections rather than mono-infections. Besides, molecular diagnostics revealed that 21% of initially reported mono-infections of *P. falciparum* were, in fact, mixed infections. No article was found on the prevalence of severe malaria or case fatality rate due to mixed or non-falciparum infections.

**Conclusion:**

A critical knowledge gap exists regarding the impact of mixed and non-falciparum species on severe malaria and death in Uganda. Robust evidence on prevalence, recurrent parasitaemia, and severe clinical manifestations of mixed and non-falciparum malaria infections is crucial for evidence-based and effective policymaking regarding malaria case management.

## Background

Reduction of malaria mortality remains among top priorities of the endemic countries. As outlined in Target 3.3 Sustainable Development Goals, the World Health Organization (WHO) member states have targeted a minimum 90% reduction in the global malaria mortality rate by 2030 compared to 2015 [1–3]. In 2022, Uganda accounted for approximately 5.1% of global malaria cases, ranked third in terms of malaria burden and eighth in malaria-attributed death worldwide [4]. The country has embraced many interventions aimed at malaria mortality reduction to less than 1 death per 100,000 population by 2030 [5].

Over the last decade, the Uganda Ministry of Health reported a 27% reduction in malaria deaths [5]. While this achievement is promising, malaria remains the largest contributor to morbidity and mortality in the country. In Uganda, severe malaria is responsible for 15–20% of hospital admissions and is the leading cause of mortality among children under 5 yearswith an estimated 18,000 malaria-attributed deaths in 2022 [4]. Inadequate access to qualified and affordable case management services, poor care-seeking behaviour [6–10], and the emergence and spread of artemisinin and partner drug resistance are important challenges that may hinder progress [11–13].

In addition, an overlooked factor may also contribute to malaria deaths. Increasing alarming signals emerged from the literature are highlighting the possibility of contribution of neglected non-falciparum malaria infections to malaria mortality. *Plasmodium falciparum* is the main malaria species that causes severe disease and malaria-attributed mortality [14]. Notwithstanding often being considered benign, growing evidence confirms severe disease and morbidity associated with *Plasmodium malariae*, *Plasmodium ovale* spp*.* and *Plasmodium vivax* infections albeit at much lower rates than *P. falciparum* infection [14–21].

Non-falciparum malaria species also can present a chronic pattern of infections with frequent recrudescences or relapses that may cause serious health complications [22–24]. Chronic infection with *P. malariae* can cause severe complications in approximately 3% of cases, including refractory nephrotic syndrome, splenomegaly, and anaemia [14, 16, 25, 26]. Literature findings indicate that patients with mixed *P. falciparum/P. malariae* infections may have a higher proportion of multiple organ failure, severe anaemia, and pulmonary complications than those with mono-infection of *P. falciparum* [20]. Besides, several studies have suggested that mixed *P. falciparum*/*P. malariae* infections were associated with increased *P. falciparum* gametocytaemia, which may accelerate malaria transmission[16, 27, 28].

One of key difference between *P. ovale* spp*.* or *P. vivax* with *P. falciparum* is the possibility of relapses [29, 30]. Without radical treatment using primaquine, the risk for relapse in *P. vivax* and *P. ovale* spp*.* is estimated to be around 33.3% and 10.0%, respectively [31]. Each recurrent episode of symptomatic malaria causes haemolysis.

Increasing evidence in many sub-Saharan African countries following introduction of molecular techniques highlights underreporting and underestimation of non-falciparum malaria species [16, 30]. This is due to the widespread use of rapid diagnostic tests (RDTs) that only detects *P. falciparum*. In addition, microscopy is not a sensitive tool for diagnosis of mixed infections due to the lower parasite density of *P. malariae*, *P. vivax* and *P. ovale* spp*.* compared with *P. falciparum* [32]. In Uganda, the proportion of suspected malaria cases who were tested using RDTs that detect only *P. falciparum* increased from 2% in 2010 to 78% in 2014, remaining above 75% from 2014 to 2022 [4].

Therefore, it is important to investigate the prevalence of non-falciparum malaria infections in Uganda, both mono and mixed infections as well as their disease severity and treatment outcome. The findings of the study will identify potential solutions to enhance malaria case management policy.

## Methods

The available English-language published peer-reviewed literature from 2005 up to March 2024 was sought via PubMed and Google Scholar. The used keywords for the search were severe malaria AND *P. falciparum*, *P. malariae*, *P. vivax*, *P. ovale* spp*.*, mixed infections AND Uganda, as well as Uganda AND *P. malariae*, *P. vivax*, *P. ovale* spp*.*, and mixed infections. After reviewing titles of the 7005 records, the authors excluded a large number of articles that were irrelevant and removed duplicate articles. In the next steps, the authors reviewed 254 abstracts of remaining articles and excluded irrelevant items. Some articles were excluded after reviewing the full content which finally left 53 articles. The selected articles were reviewed on three main themes including (1) prevalence of non-falciparum malaria infections in Uganda and (2) Clinical manifestations of severe malaria in non-falciparum infections in Uganda, and (3) Non-falciparum malaria case management in Uganda. Given the limitation of RDTs and microscopy methods in diagnosis of non-falciparum species, studies using molecular methods have been used to estimate the prevalence of non-falciparum malaria infections and clinical manifestation of severe malaria in non-falciparum infections in Uganda. The article is structured around these three key themes. To enrich the discussion, the authors searched WHO website for relevant WHO reports and technical documents.

## Results and discussion

### Prevalence of *Plasmodium* species in Uganda

Five studies that used molecular tests for diagnosis of malaria species have been selected for further analysis of the prevalence of *Plasmodium* species in Uganda. The characteristics of the studies included in the review are presented in Table 1 and the included studies results are presented in Table 2.

**Table 1:** Characteristics of the studies included in the review

No	Author/year	Year	Study design	Method of malaria detection	Number of Samples	Participants	Participants Age range	Geographic areas	Total malaria
1	Betson et al. [58]	2014	Community-based longitudinal study	Real-time polymerase chain reaction	163	Confirmed malaria by RDT or microscopy	2–7	Nationwide	163
2	Asua et al. [59]	2017	Quantitative study	Nested polymerase chain reaction	474	Confirmed malaria by RDT or microscopy	6 m–10 Y	Nationwide	474
3	Betson et al. [16]	2018	The longitudinal, closed-cohort study	Real-time polymerase chain reaction	1211	General population	5 m–6 Y	Albert and Victoria Lakeshore	896
					662		15–60		250
4	Subissi et al. [32]	2019	Cross-sectional surveys	Nested polymerase chain reaction	249	General population	1–5	Apac	145
					126		6–10		111
					134		> 20		34
5	Murphy et al. [35]	2020	Quantitative study	Nested polymerase chain reaction	1000	Asymptomatic blood donors	> 17	Kampala and Jinja	154

**Table 2:** Prevalence of *Plasmodium* species in Uganda using molecular testing

Author/year	Sample	Inclusion	Age	Prevalence (%)					Details of mixed infections species	Non-F %*
				*P.f*%	*P.m*.%	*P.o*%	*P.v*%	Mixed %		
Betson et al. [58]	163	Confirmed malaria by RDT or microscopy	2–7	100				58	Mixed 58% (*Pf* + *Pm* 41%, *Pf* + *Po* 9%, and three species 8%)	58
Asua et al. [59]	474	Confirmed malaria by RDT or microscopy	6 m-10Y	91.8				8.6	*Pf* + *Pm* 4.6%, *Pf* + *Po*3.2%, *Pf* + *Pv* 0.84%, one case three species *Pm* + *Pf* + *Po*	8.6
Betson et al. [16]	1211	General population	5 m -6 Y	74	7.4	2.8			The majority of children infected with *Pm* and *Po* were also infected with *Pf*	14
	662		15- 60	37.7					Only 9 mothers were infected with *Pm*, and only 2 were infected with*.o*	4
Subissi et al. [32]	249	General population	1–5	40.6	2.2	1.3	0	14	*Pf* + *Pm* 7.9%, *Pf* + *Po* 3.9%*, Pf* + *Po* + *Pm* 2.2%	31
	126		6–10	55	1	3	0	29	*Pf* + *Pm* 19%, *Pf* + *Po* 5%, *Pf* + *Po* + *Pm* 5%	38
	134		> 20	21.3	0.9	1.7	0	1.7	*Pf* + *P.m*1.7%	16
Murphy et al. [35]	1000	Asymptomatic blood donors	> 17	9.3	1.3	0.5	0	4.3	*Pf* + *Pm* 2.3%, *Pf* + *Po* 0.9%, *Pf* + *Po* + *Pm* 1.1%	40

**Fig. 1:**
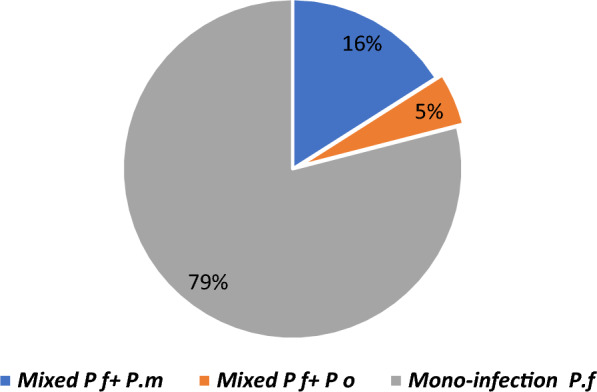
Pooled analysis of initially diagnosed mono *P. falciparum* infection

**Fig. 2:**
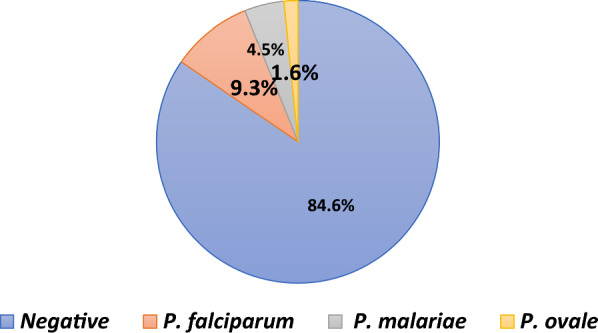
Proportion of confirmed *Plasmodium* in asymptomatic blood donors

Overall pooled analysis of *Plasmodium* species in 2227 positive cases in 4019 samples showed:

Non-falciparum malaria:

- Molecular diagnostics revealed that 21% of initially reported mono-infections of *P. falciparum* were, in fact, mixed infections, *P. falciparum/P. malariae* 16%, and *P. falciparum/P. ovale* spp*.* 5% (Fig. 1).
- Non-falciparum infections were more common as coinfections with *P. falciparum* rather than mono-infection.
- Mixed infections of three species (*P. falciparum*, *P. malariae*, and *P. ovale* spp*.*) were rarely reported.
- *P. malariae* was the second most prevalent species (9.7% of positive cases infected by *P. malariae* species, mono or mixed infections with *P. falciparum*)
- *P. ovale* spp*.* was the third most prevalent species (4% of positive cases infected by *P. ovale* spp*.* species, mono or mixed infections with *P. falciparum*)
- *P. vivax* was rarely reported. Other studies reported *P. vivax* in Uganda as well [33, 34].
- A significant heterogeneity in the prevalence of non-falciparum malaria infections was found in different geographical areas and among various age groups. The range of non-falciparum infections varied from 4 to 58% of positive cases. On average non-falciparum infections accounted for 18.6% of positive cases.
- Results of study of Murphy in the blood donors revealed that 9.3%, 4.5%, 1.6% asymptomatic cases were infected with *P. falciparum*, *P. malariae* and, *P. ovale* spp*.,* respectively [35] (Fig. 2).

The findings of this review are similar to the results of studies in other countries. The prevalence of *P. malariae* and the total prevalence of *P. malariae* and *P. ovale* spp*.* in sub-Saharan Africa has been estimated at around 10% [23] and 20% [30], respectively. A higher prevalence of mixed infections of non-falciparum malaria compared with their mono-infections has been confirmed in other countries as well [16, 30]. The presence of *P. malariae* and *P. ovale* spp*.* both mono and mixed infections in Uganda using microscopy has been confirmed [16, 27, 36–38]. Besides, the transmission patterns of the non-falciparum species do not necessarily follow those of *P*. *falciparum*, stressing the need for attention towards non-falciparum malaria in Africa [39].

### Clinical manifestations of severe malaria in non-falciparum infections in Uganda

The authors did not find any articles using molecular methods investigating the impact of non-falciparum malaria infections on disease severity and malaria mortality in Uganda. The reviewed literature on severe malaria and the majority of articles on uncomplicated malaria in Uganda relied on RDTs or light microscopy [5, 8–10, 14, 20, 29, 40–49]. Non-falciparum malaria prevalence may have been underestimated in studies relying on rapid diagnostic tests (RDTs) detecting only *P. falciparum* [14, 27, 32, 42, 50].

Underestimation of non-falciparum malaria infections may happen using microscopy-based methods as well. Diagnosis of *P. malariae* and *P. ovale* spp*.* both mixed and mono-infections by light microscopy can be difficult because non-falciparum parasitaemias often occur below detection thresholds or are masked by more visible, concurrent *P. falciparum* species [14, 16, 32, 42, 51, 52]. The similarity of *P. malariae* and *P. falciparum* parasites, microscopists competency, and laboratory infrastructure are other factors affecting the accuracy of diagnosis of malaria species and mixed infections by microscopy [14, 42].

### Non-falciparum malaria case management in Uganda

Malaria case management policy in many sub-Saharan countries including Uganda has focused on *P. falciparum* [14]. The policy has been set based on this argument that non-falciparum malaria infections are mild and easily curable with common anti-malarial medicines recommended for *P. falciparum*.

The review found one article in Uganda that its results indicated persistent chronic multi-species malaria infections (9.2%) in children after artemether/lumefantrine treatment [16] indicating this hypothesis that artemether/lumefantrine may not be an effective medicine to treat *P. malariae*.

In addition, alarming literature evidence in other countries has been found on the possibility of treatment failure of non-falciparum malaria infections following treatment with mefloquine, halofantrine, quinine, and artemisinin-based combination [14, 24, 52]. A significant reduction in ex vivo susceptibility of *P. malariae* to lumefantrine and artemether has been reported in Mali [42].

It should be considered that recurrent episodes can occur due to recrudescence, relapse (in *P. vivax* and *P. ovale* spp*.* infections), or a new infection [17, 53]. Therefore, without robust evidence, recurrent episodes cannot be considered equal to reinfection.

**Fig. 3:**
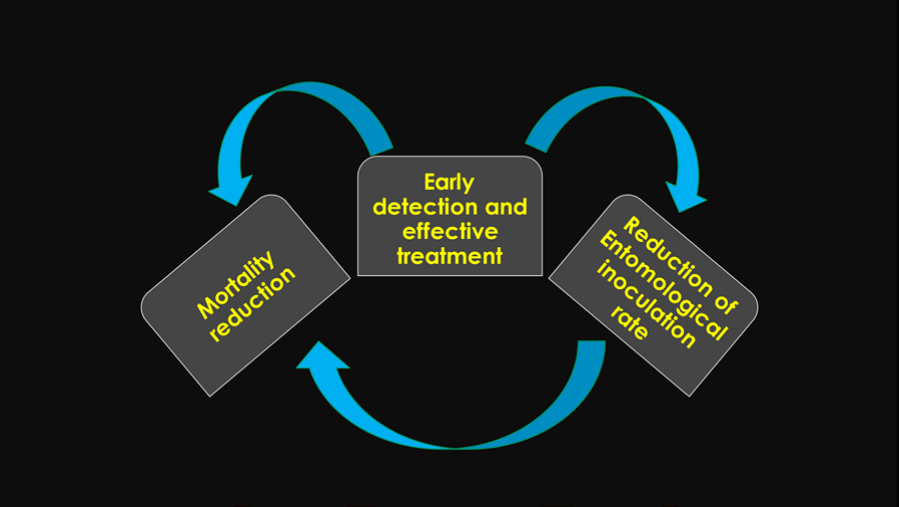
Importance of early detection and effective treatment of all malaria cases

The WHO emphasizes that the programme should ensure access to early diagnosis and prompt, effective treatment [54–56]. Strong surveillance, case detection, diagnosis, and treatment have direct benefits in reducing mortality and severe malaria disease but additionally can reduce transmission by diminishing the pool of infected individuals, which in return indirectly reduces malaria mortality [8, 17, 55] (Fig. 3). This recommendation covers all malaria species.

To address concerns regarding *P. malariae* treatment response, in some studies, using artemisinin combination therapy with a long half-life partner drug was recommended [16, 24]. The published evidence in the literature is insufficient to conclude common antimalarial medicines recommended for *P. falciparum* are not effective for non-falciparum malaria treatment in Uganda. Given any changes in case management policy will have policy implications, should be justified by robust evidence, and its feasibility and its pros and cons should be carefully considered.

Regarding diagnosis methods of suspected severe malaria cases, some studies emphasized the importance of diagnosis of suspected severe malaria cases due to infections by all malaria species including mono and mixed non-falciparum infections as well as awareness raising of physicians regarding the possibility of severe disease of neglected species in areas where more than one species is prevalent [19, 20, 26]. The WHO recommendations highlight that RDT can be used to confirm malaria rapidly however, microscopy is preferred for diagnosis of severe malaria as in addition to diagnosis species, it can provide other important parameters of prognostic relevance [54]. If quality-assured microscopy services are provided in hospitals of endemic countries, mixed infections as well as mono infections of non-falciparum malaria infections, particularly in severe malaria cases can be detected.

In the reviewed articles the authors didn’t find any published paper covering radical treatment of *P. ovale* spp*.* or *P. vivax* in Uganda. To prevent relapse in malaria cases infected by *P. ovale* spp*.* or *P. vivax*, the WHO recommends radical treatment in all transmission settings (except for those that have contraindications of primaquine) [57].

## Conclusion

Non-falciparum malaria infections are neglected malaria species in sub-Saharan countries including Uganda where *P. falciparum* is the dominant species and the main cause of severe disease and mortality. This caused a knowledge gap in epidemiology, biology, health impact, and the role of mixed or mono-infections of non-falciparum species particularly regarding severe forms of malaria. Given mixed infection is common in Uganda, further research using reliable malaria species diagnosis methods to address this gap is recommended.

Besides, the focus of case management in Uganda is on *P. falciparum*. The policy has been set based on this argument that non-falciparum malaria infections are mild and easily curable with common antimalarial medicine recommended for *P. falciparum*. There is insufficient evidence in the literature on treatment outcomes of non-falciparum malaria of mixed infections to challenge this policy.

Finally, the health workforce in high-endemic countries where non-falciparum infections are common should be informed that mixed and mono-infections of non-falciparum malaria species can be seen. This may save the life of severe malaria cases with negative RDTs or severe malaria cases with frequent recurrent parasitaemia after discharge when its reason may not be reinfection of *P. falciparum*.

## Data Availability

Data sharing is not applicable to this article as no datasets were generated during the current study.

## Abbreviations

RDT: Rapid diagnostic tests
*Pf*: *Plasmodium falciparum*
*Pm*: *Plasmodium malaria*
*Pv*: *Plasmodium vivax*
*Po*: *Plasmodium ovale* Spp*.*
WHO: World Health Organization
